# On bounds involving *k*-Appell’s hypergeometric functions

**DOI:** 10.1186/s13660-017-1391-2

**Published:** 2017-05-18

**Authors:** Muhammad Uzair Awan, Muhammad Aslam Noor, Marcela V Mihai, Khalida Inayat Noor

**Affiliations:** 1 Department of Mathematics, Government College University, Faisalabad, Pakistan; 2 Department of Mathematics, King Saud University, Riyadh, Saudi Arabia; 3 Department of Mathematics, COMSATS Institute of Information Technology, Islamabad, Pakistan; 4 Department scientific-methodical sessions, Romanian Mathematical Society-branch Bucharest, Academy Street no. 14, Bucharest, 010014 Romania

**Keywords:** 26D15, 26A51, 33B15, 33C65, convex functions, harmonic convex functions, *k*-fractional, *k*-Appell’s hypergeometric functions, inequalities

## Abstract

In this paper, we derive a new extension of Hermite-Hadamard’s inequality via *k*-Riemann-Liouville fractional integrals. Two new *k*-fractional integral identities are also derived. Then, using these identities as an auxiliary result, we obtain some new *k*-fractional bounds which involve *k*-Appell’s hypergeometric functions. These bounds can be viewed as new *k*-fractional estimations of trapezoidal and mid-point type inequalities. These results are obtained for the functions which have the harmonic convexity property. We also discuss some special cases which can be deduced from the main results of the paper.

## Introduction and preliminaries

Convexity theory has played a pivotal role through its numerous applications in different fields of pure and applied sciences. In the past few years several new generalizations and extensions of classical convexity have been proposed in the literature, see [1–12]. Shi et al. [11] introduced the notion of harmonic convex sets as follows.

### Definition 1.1

[11]

A set $\Omega\subset\mathbb{R}_{+}$ is said to be a harmonically convex set if

$$\begin{aligned} \frac{xy}{tx+(1-t)y}\in\Omega,\quad\forall x,y\in\Omega,t\in[0,1]. \end{aligned}$$

Iscan [8] introduced the class of harmonic convex functions. The natural domain of harmonic convex functions is harmonic convex sets. Noor et al. [10] extended the definition of harmonic convex functions and defined a new generalization, which is called harmonic *h*-convex functions.

### Definition 1.2

[10]

Let $h:[0,1]\subseteq J\rightarrow\mathbb{R}$ be a real function. A function $f:\Omega\subset\mathbb{R}_{+}\rightarrow\mathbb{R}$ is said to be a harmonically *h*-convex function if

1.1 $$\begin{aligned} f \biggl(\frac{xy}{tx+(1-t)y} \biggr)\leq h(1-t)f(x)+h(t)f(y),\quad \forall x,y\in I, t\in(0,1). \end{aligned}$$

### Remark 1.3

Note that, if $h(t)=t,t^{s},t^{-s},t^{-1}$ and $t=1$, then the definition of harmonic *h*-convex functions reduces to the definitions of harmonic convex, harmonic *s*-convex, harmonic *s*-Godunova-Levin convex, harmonic Godunova-Levin and harmonic *P*-functions, respectively. Thus it is worth to mention here that the class of harmonic *h*-convex functions is quite unifying one as it naturally includes several other classes of harmonic convex functions.

Convexity theory has also a strong relationship with theory of inequalities, and resultantly many inequalities have been obtained via convex functions, see [6, 13–15]. Interested readers may find the importance of generalized convexity to variational inequalities and multiple objective optimization in [16–20]. One of the most extensively studied inequalities is Hermite-Hadamard’s inequality. This inequality was proved by Hermite and Hadamard independently. It provides a necessary and sufficient condition for a function to be convex. Dragomir et al. [6] has written a nice monograph on Hermite-Hadamard type inequalities. Interested readers may find very interesting and useful details about these inequalities in this monograph. Khattri [21] discussed some very interesting applications of Hermite-Hadamard’s inequality. Recently fractional calculus has attracted many researchers and thus become a powerful tool in many branches of mathematics. For some recent investigations in fractional calculus, see [22]. The classical form of Riemann-Liouville integrals is defined as follows.

### Definition 1.4

[22]

Let $f\in L_{1}[a,b]$. Then the Riemann-Liouville integrals $J_{a^{+}}^{\alpha}f$ and $J_{b^{-}}^{\alpha}f$ of order $\alpha>0$ with $a\geq0$ are defined by

1.2 $$\begin{aligned} J_{a^{+}}^{\alpha}f(x)=\frac{1}{\Gamma(\alpha)} \int_{a}^{x}(x-t)^{\alpha-1}f(t)\,\mathrm {d}t,\quad x>a, \end{aligned}$$

and

1.3 $$\begin{aligned} J_{b^{-}}^{\alpha}f(x)=\frac{1}{\Gamma(\alpha)} \int_{x}^{b}(t-x)^{\alpha-1}f(t)\,\mathrm {d}t,\quad x< b, \end{aligned}$$

where

$$\begin{aligned} \Gamma(\alpha)= \int_{0}^{\infty}e^{-t}x^{\alpha-1}\, \mathrm {d}x, \end{aligned}$$

is the well-known gamma function.

Sarikaya et al. [23] obtained Hermite-Hadamard type inequalities via Riemann-Liouville fractional integrals. Diaz et al. [24] introduced the generalized *k*-gamma function as

1.4 $$\begin{aligned} \Gamma_{k}(x)=\lim_{n\rightarrow\infty}\frac{n!k^{n}(nk)^{\frac {x}{k}-1}}{(x)_{n,k}},\quad k>0, x\in\mathbb{C}\setminus k\mathbb{Z}^{-}. \end{aligned}$$

$\Gamma_{k}$ is one parameter deformation of the classical gamma function as $\Gamma_{k}\rightarrow\Gamma$ when $k\rightarrow1$. $\Gamma_{k}$ is based on the repeated appearance of the expression of

$$\phi(\phi+k) (\phi+2k) (\phi+3k)\cdots \bigl(\phi+(n-1)k \bigr). $$

This above statement is a function of the variable *ϕ* and is denoted by $(\phi)_{n,k}$. It is known as Pochhammer *k*-symbol, which reduces to classical Pochhammer symbol $(\phi)_{n}$ by taking $k=1$. The integral of $\Gamma_{k}$ is given by

1.5 $$\begin{aligned} \Gamma_{k}(x)= \int_{0}^{\infty}t^{x-1}e^{-\frac{t^{k}}{k}} \,\mathrm {d}t,\quad\Re(x)>0. \end{aligned}$$

It is evident from (1.5) that

$$\Gamma_{k}(x)=k^{\frac{x}{k}-1}\Gamma \biggl(\frac{x}{k} \biggr). $$

Diaz et al. [24] also defined a *k*-beta function as

1.6 $$\begin{aligned} \beta_{k}(x,y)=\frac{\Gamma_{k}(x)\Gamma_{k}(y)}{\Gamma_{k}(x+y)}, \quad\Re(x)>0, \Re(y)>0. \end{aligned}$$

The integral form of a *k*-beta function is given by

1.7 $$\begin{aligned} \beta_{k}(x,y)=\frac{1}{k} \int_{0}^{1}t^{\frac{x}{k}-1}(1-t)^{\frac{x}{k}-1} \, \mathrm {d}t. \end{aligned}$$

From (1.5) and (1.7) one can have

$$\beta_{k}(x,y)=\frac{1}{k}\beta \biggl(\frac{x}{k}, \frac{y}{k} \biggr). $$

Using these definitions of *k*-gamma and *k*-beta functions, Mubeen et al. [25] introduced the *k*-Riemann-Liouville fractional integral of the type

1.8 $$\begin{aligned} {}_{k}J^{\alpha}f(x)=\frac{1}{k\Gamma_{k}(\alpha)} \int_{0}^{x}(x-t)^{\frac{\alpha}{k}-1}f(t)\,\mathrm {d}t, \quad \alpha>0,x>0,k>0. \end{aligned}$$

It is obvious that when $k\rightarrow1$, the above definition reduces to classical Riemann-Liouville fractional integrals.

Sarikaya et al. [26] introduced the notion of *k*-Riemann-Liouville fractional integrals and discussed some of its interesting applications with respect to inequalities.

To be more precise, let *f* be piecewise continuous on $I^{*}=(0,\infty)$ and integrable on any finite subinterval of $I=[0,\infty]$. Then, for $t>0$, we consider the *k*-Riemann-Liouville fractional integral of *f* of order *α*

$$\begin{aligned} _{k}J_{a}^{\alpha}f(x)=\frac{1}{k\Gamma_{k}(\alpha)} \int_{a}^{x}(x-t)^{\frac{\alpha}{k}-1}f(t)\,\mathrm {d}t, \quad x>a,k>0. \end{aligned}$$

For more details, see [26]. Note that when $k\rightarrow1$, *k*-Riemann-Liouville fractional integrals become classical Riemann-Liouville fractional integrals. It is worth mentioning here that the notion of *k*-Riemann-Liouville fractional integral is the significant generalization of all above Riemann-Liouville fractional integrals. We would like to emphasize that for $k\neq1$ the properties of *k*-Riemann-Liouville fractional integrals are quite different from those of classical Riemann-Liouville fractional integrals. Due to these facts, the *k*-Riemann-Liouville fractional integrals have important applications in several branches of pure and applied sciences, see [24, 26, 27].

The integral representation of *k*-Appell’s series $F_{1,k}$, where $k>0$, is

$$\begin{aligned} F_{1,k}=\frac{\Gamma_{k}(c)}{k\Gamma_{k}(a^{\prime})\Gamma _{k}(c-a^{\prime})} \int_{0}^{1}t^{\frac{a^{\prime}}{k}-1}(1-t)^{\frac{c-a^{\prime }}{k}-1}(1-kz_{1}t)^{-\frac{b_{1}}{k}}(1-kz_{2}t)^{-\frac{b_{2}}{k}} \,\mathrm {d}t. \end{aligned}$$

For some more details, see [27].

## Some new auxiliary results

In this section, we derive some new *k*-fractional identities which will serve as auxiliary results for the developments of our next results.

### Lemma 2.1

*Let* $f:I\setminus\{0\}\to\mathbb{R}$ *be differentiable on* $I^{\circ}$ *such that* $f'\in L[a,b]$, *where* $a,b\in I$ *with* $a< b$, *then*

$$\begin{aligned} T_{f}(a,b;\alpha,k;g) &=\frac{ab(b-a)}{2} \int_{0}^{1}\frac{[t^{\frac{\alpha}{k}}-(1-t)^{\frac{\alpha }{k}}]}{[ta+(1-t)b]^{2}}f' \biggl(\frac{ab}{ta+(1-t)b} \biggr)\,\mathrm {d}t, \end{aligned}$$

*where*

$$\begin{aligned} &{T_{f}(a,b;\alpha,k;g)} \\ &{\quad =\frac{f(a)+f(b)}{2}-\frac{\Gamma_{k}(\alpha+k)}{2} \biggl(\frac{ab}{b-a} \biggr)^{\frac{\alpha}{k}} \biggl[ {}_{k}J_{\frac{1}{b^{+}}}^{\alpha}(f \circ g) \biggl(\frac{1}{a} \biggr)+ {}_{k}J_{\frac{1}{a^{-}}}^{\alpha}(f \circ g) \biggl(\frac{1}{b} \biggr) \biggr].} \end{aligned}$$

### Proof

It suffices to show that

2.1 $$\begin{aligned} T_{f}(a,b;\alpha,k;g) =&\frac{ab(b-a)}{2} \int_{0}^{1}\frac{[t^{\frac{\alpha}{k}}-(1-t)^{\frac{\alpha }{k}}]}{[ta+(1-t)b]^{2}}f' \biggl(\frac{ab}{ta+(1-t)b} \biggr)\,\mathrm {d}t \\ =& K_{1}+K_{2}. \end{aligned}$$

Now integrating by parts yields

2.2 $$\begin{aligned} K_{1}&=\frac{ab(b-a)}{2} \int_{0}^{1}\frac{t^{\frac{\alpha}{k}}}{[ta+(1-t)b]^{2}}f' \biggl(\frac{ab}{ta+(1-t)b} \biggr)\,\mathrm {d}t \\ &=\frac{1}{2} \biggl[f(b)-\frac{k\Gamma_{k}(\alpha+k)}{k} \biggl(\frac{ab}{b-a} \biggr)^{\frac{\alpha}{k}}\frac{1}{k\Gamma_{k}(\alpha)} \int_{\frac{1}{b}}^{\frac{1}{a}} \biggl(\frac{1}{a}-x \biggr)^{\frac{\alpha}{k}-1}f \biggl(\frac{1}{x} \biggr)\,\mathrm {d}x \biggr] \\ &=\frac{f(b)}{2}-\frac{\Gamma_{k}(\alpha+k)}{2} \biggl(\frac{ab}{b-a} \biggr)^{\frac{\alpha}{k}} {}_{k}J_{\frac{1}{b^{+}}}^{\alpha}(f\circ g) \biggl(\frac{1}{a} \biggr). \end{aligned}$$

Similarly

2.3 $$\begin{aligned} K_{2}&=\frac{ab(b-a)}{2} \int_{0}^{1}\frac{(1-t)^{\frac{\alpha}{k}}}{[ta+(1-t)b]^{2}}f' \biggl(\frac{ab}{ta+(1-t)b} \biggr)\,\mathrm {d}t \\ &=\frac{f(a)}{2}-\frac{\Gamma_{k}(\alpha+k)}{2} \biggl(\frac{ab}{b-a} \biggr)^{\frac{\alpha}{k}} {}_{k}J_{\frac{1}{a^{-}}}^{\alpha}(f\circ g) \biggl(\frac{1}{b} \biggr). \end{aligned}$$

Combining (2.1), (2.2) and (2.3) completes the proof. □

### Lemma 2.2

*Under the assumptions of Lemma* 2.1 *and* $k=1$, *we have*

$$\begin{aligned} T_{f}(a,b;\alpha,1;g) &=\frac{ab(b-a)}{2} \int_{0}^{1}\frac{t^{\alpha}-(1-t)^{\alpha}}{[ta+(1-t)b]^{2}}f' \biggl(\frac{ab}{ta+(1-t)b} \biggr)\,\mathrm {d}t, \end{aligned}$$

*where*

$$T_{f}(a,b;\alpha,1;g) =\frac{f(a)+f(b)}{2}-\frac{\Gamma(\alpha+1)}{2} \biggl(\frac{ab}{b-a} \biggr)^{\alpha} \biggl[ J_{\frac{1}{b^{+}}}^{\alpha}(f\circ g) \biggl( \frac{1}{a} \biggr)+ J_{\frac{1}{a^{-}}}^{\alpha}(f\circ g) \biggl( \frac{1}{b} \biggr) \biggr]. $$

This is due to Iscan [8].

### Lemma 2.3

*Let* $f:I\setminus\{0\}\to\mathbb{R}$ *be differentiable on* $I^{\circ}$ *such that* $f'\in L[a,b]$, *where* $a,b\in I$ *with* $a< b$, *then*

$$\begin{aligned} M_{f}(a,b;\alpha,k;g) =&\frac{1}{2}\sum _{i=1}^{3}I_{i} \\ =&\frac{1}{2} \biggl[ ab(b-a) \int_{0}^{\frac{1}{2}}\frac{1}{[ta+(1-t)b]^{2}}f' \biggl(\frac{ab}{ta+(1-t)b} \biggr)\,\mathrm {d}t \\ &{}-ab(b-a) \int_{\frac{1}{2}}^{1}\frac{1}{[ta+(1-t)b]^{2}}f' \biggl(\frac{ab}{ta+(1-t)b} \biggr)\,\mathrm {d}t \\ &{}-ab(b-a) \int_{0}^{1} \bigl[(1-t)^{\frac{\alpha}{k}}-t^{\frac{\alpha}{k}} \bigr]\frac{1}{[ta+(1-t)b]^{2}}f' \biggl(\frac{ab}{ta+(1-t)b} \biggr)\, \mathrm {d}t \biggr] , \end{aligned}$$

*where*

$$\begin{aligned} &{M_{f}(a,b;\alpha,k;g)} \\ &{\quad=f \biggl(\frac{2ab}{a+b} \biggr)-\frac{\Gamma_{k}(\alpha+1)}{2} \biggl( \frac{ab}{b-a} \biggr)^{\frac{\alpha}{k}} \biggl\{ {}_{k}J_{\frac {1}{b^{-}}}^{\alpha}(f \circ g) \biggl( \frac{1}{a} \biggr) + {}_{k}J_{\frac{1}{a^{+}}}^{\alpha}(f \circ g) \biggl( \frac{1}{b} \biggr) \biggr\} .} \end{aligned}$$

### Proof

Calculate $I_{1}$, $I_{2}$ and $I_{3}$ as follows:

2.4 $$\begin{aligned} I_{1}={}&ab(b-a) \int_{0}^{\frac{1}{2}}\frac{1}{[ta+(1-t)b]^{2}}f' \biggl(\frac{ab}{ta+(1-t)b} \biggr)\,\mathrm {d}t \\ ={}&f \biggl(\frac{2ab}{a+b} \biggr)-f(a). \end{aligned}$$

Now

2.5 $$\begin{aligned} I_{2}={}&-ab(b-a) \int_{\frac{1}{2}}^{1}\frac{1}{[ta+(1-t)b]^{2}}f' \biggl(\frac{ab}{ta+(1-t)b} \biggr)\,\mathrm {d}t \\ ={}&f \biggl(\frac{2ab}{a+b} \biggr)-f(b). \end{aligned}$$

Also

2.6 $$\begin{aligned} I_{3}={}&-ab(b-a) \int_{0}^{1} \bigl[(1-t)^{\frac{\alpha}{k}}-t^{\frac{\alpha}{k}} \bigr]\frac{1}{[ta+(1-t)b]^{2}}f' \biggl(\frac{ab}{ta+(1-t)b} \biggr)\, \mathrm {d}t \\ ={}&- \int_{0}^{1} \bigl[(1-t)^{\frac{\alpha}{k}}-t^{\frac{\alpha}{k}} \bigr]\,\mathrm {d}f \biggl(\frac{ab}{ta+(1-t)b} \biggr) \\ ={}&- \int_{0}^{1}(1-t)^{\frac{\alpha}{k}}\,\mathrm {d}f \biggl( \frac{ab}{ta+(1-t)b} \biggr)+ \int_{0}^{1}t^{\frac{\alpha}{k}}\,\mathrm {d}f \biggl( \frac{ab}{ta+(1-t)b} \biggr) \\ ={}&I_{I}+I_{II}. \end{aligned}$$

Now consider

$$\begin{aligned} I_{I}={}&- \int_{0}^{1}(1-t)^{\frac{\alpha}{k}}\,\mathrm {d}f \biggl( \frac{ab}{ta+(1-t)b} \biggr) \\ ={}&f(a)-\frac{\alpha}{k} \int_{0}^{1}(1-t)^{\frac{\alpha}{k}-1}f \biggl( \frac{ab}{ta+(1-t)b} \biggr)\,\mathrm {d}t. \end{aligned}$$

Now suppose $u=\frac{ab}{ta+(1-t)b}$, then

$$\begin{aligned} I_{I}={}&f(a)-\frac{\alpha}{k} \biggl(\frac{ab}{b-a} \biggr)^{\frac{\alpha}{k}} \int_{a}^{b} \biggl(\frac{1}{u}- \frac{1}{b} \biggr)^{\frac{\alpha}{k}-1}\frac{1}{u^{2}}f(u)\,\mathrm {d}u. \end{aligned}$$

Again suppose $u=\frac{1}{t}$, then

2.7 $$\begin{aligned} I_{I}={}&f(a)-\Gamma_{k}(\alpha+k) \biggl( \frac{ab}{b-a} \biggr)^{\frac{\alpha}{k}} {}_{k}J_{\frac {1}{b^{-}}}^{\alpha}(f \circ g) \biggl( \frac{1}{a} \biggr) . \end{aligned}$$

Similarly

2.8 $$\begin{aligned} I_{II} ={}& f(b)-\Gamma_{k}(\alpha+k) \biggl( \frac{ab}{b-a} \biggr)^{\frac{\alpha}{k}} {}_{k}J_{\frac {1}{a^{+}}}^{\alpha}(f \circ g) \biggl( \frac{1}{b} \biggr) . \end{aligned}$$

Using (2.7) and (2.8) in (2.6) and then adding the resultant with (2.4) and (2.5) completes the proof. □

### Lemma 2.4

*Under the assumptions of Lemma* 2.3, *if* $k\to1$, *we have*

$$\begin{aligned} M_{f}(a,b;\alpha,1;g) =&\frac{1}{2}\sum _{i=1}^{3}I_{i} \\ =&\frac{1}{2} \biggl[ ab(b-a) \int_{0}^{\frac{1}{2}}\frac{1}{[ta+(1-t)b]^{2}}f' \biggl(\frac{ab}{ta+(1-t)b} \biggr)\,\mathrm {d}t \\ &{} -ab(b-a) \int_{\frac{1}{2}}^{1}\frac{1}{[ta+(1-t)b]^{2}}f' \biggl(\frac{ab}{ta+(1-t)b} \biggr)\,\mathrm {d}t \\ &{}-ab(b-a) \int_{0}^{1} \bigl[(1-t)^{\alpha}-t^{\alpha} \bigr]\frac{1}{[ta+(1-t)b]^{2}}f' \biggl(\frac{ab}{ta+(1-t)b} \biggr)\, \mathrm {d}t \biggr] , \end{aligned}$$

*where*

$$M_{f}(a,b;\alpha,1;g) =f \biggl(\frac{2ab}{a+b} \biggr)-\frac{\Gamma(\alpha+1)}{2} \biggl( \frac{ab}{b-a} \biggr)^{\frac{\alpha}{k}} \biggl\{ J_{\frac {1}{b^{-}}}^{\alpha}(f \circ g) \biggl( \frac{1}{a} \biggr) + J_{\frac{1}{a^{+}}}^{\alpha}(f \circ g) \biggl( \frac{1}{b} \biggr) \biggr\} . $$

This result is due to Set et al. [28].

## Results and discussions

In this section, we derive some new *k*-fractional integral inequalities.

### Theorem 3.1

*Let* $f:I\setminus\{0\}\rightarrow\mathbb{R}$ *be a harmonically* *h*-*convex function where* $a,b\in I$ *with* $a< b$. *If* $f\in L[a,b]$, *then*, *for* $h (\frac{1}{2} )\neq0$, *we have*

$$\begin{aligned} \frac{k}{\alpha h(\frac{1}{2})}f \biggl(\frac{2ab}{a+b} \biggr) \leq& \biggl(\frac{ab}{b-a} \biggr)^{\frac{\alpha}{k}}k\Gamma_{k}( \alpha) \biggl\{ {}_{k}J_{\frac{1}{a^{-}}}^{\alpha}(f\circ g) \biggl( \frac{1}{b} \biggr) + {}_{k}J_{\frac{1}{b^{+}}}^{\alpha}(f\circ g) \biggl(\frac{1}{a} \biggr) \biggr\} \\ \leq& \bigl[f(a)+f(b) \bigr] \int_{0}^{1}t^{\frac{\alpha}{k}-1} \bigl[h(1-t)+h(t) \bigr] \,\mathrm {d}t. \end{aligned}$$

### Proof

Since *f* is a harmonically *h*-convex function, so we have

$$\begin{aligned} f \biggl(\frac{2ab}{(1-t)a+tb} \biggr)\leq h \biggl(\frac{1}{2} \biggr) \biggl[f \biggl(\frac{ab}{ta+(1-t)b} \biggr)+f \biggl(\frac {ab}{(1-t)a+tb} \biggr) \biggr]. \end{aligned}$$

Multiplying both sides of the above inequality by $t^{\frac{\alpha}{k}-1}$ and integrating it with respect to *t* on $[0,1]$, we have

$$\begin{aligned} &{\frac{k}{\alpha}f \biggl(\frac{2ab}{a+b} \biggr)} \\ &{\quad =f \biggl(\frac{2ab}{a+b} \biggr) \int_{0}^{1}t^{\frac{\alpha}{k}-1}\,\mathrm {d}t} \\ &{\quad \leq h \biggl(\frac{1}{2} \biggr) \biggl[ \int_{0}^{1}t^{\frac{\alpha}{k}-1}f \biggl( \frac{ab}{ta+(1-t)b} \biggr)\,\mathrm {d}t + \int_{0}^{1}t^{\frac{\alpha}{k}-1}f \biggl( \frac{ab}{(1-t)a+tb} \biggr)\,\mathrm {d}t \biggr]} \\ &{\quad =h \biggl(\frac{1}{2} \biggr) \biggl(\frac{ab}{b-a} \biggr)^{\frac{\alpha}{k}} \biggl\{ \int_{\frac{1}{b}}^{\frac{1}{a}} \biggl(x-\frac{1}{b} \biggr)^{\frac{\alpha}{k}-1} f \biggl(\frac{1}{x} \biggr)\,\mathrm {d}x+ \int_{\frac{1}{b}}^{\frac{1}{a}} \biggl(\frac{1}{a}-x \biggr)^{\frac{\alpha}{k}-1} f \biggl(\frac{1}{x} \biggr)\,\mathrm {d}x \biggr\} } \\ &{\quad =h \biggl(\frac{1}{2} \biggr) \biggl(\frac{ab}{b-a} \biggr)^{\frac{\alpha}{k}}k\Gamma_{k}(\alpha) \biggl\{ {}_{k}J_{\frac {1}{a^{-}}}^{\alpha}(f \circ g) \biggl(\frac{1}{b} \biggr) + {}_{k}J_{\frac{1}{b^{+}}}^{\alpha}(f \circ g) \biggl(\frac{1}{a} \biggr) \biggr\} .} \end{aligned}$$

This implies

3.1 $$\begin{aligned} \frac{k}{\alpha h (\frac{1}{2} )}f \biggl(\frac{2ab}{a+b} \biggr) \leq \biggl( \frac{ab}{b-a} \biggr)^{\frac{\alpha}{k}}k\Gamma_{k}(\alpha) \biggl\{ {}_{k}J_{\frac{1}{a^{-}}}^{\alpha}(f\circ g) \biggl( \frac{1}{b} \biggr) + {}_{k}J_{\frac{1}{b^{+}}}^{\alpha}(f\circ g) \biggl( \frac{1}{a} \biggr) \biggr\} . \end{aligned}$$

Now

$$\begin{aligned} &{f \biggl(\frac{ab}{ta+(1-t)b} \biggr)\leq h(1-t)f(a)+h(t)f(b),} \\ &{f \biggl(\frac{ab}{(1-t)a+tb} \biggr)\leq h(t)f(a)+h(1-t)f(b).} \end{aligned}$$

Adding the above two inequalities and multiplying both sides by $t^{\frac{\alpha}{k}-1}$, we have

$$\begin{aligned} t^{\frac{\alpha}{k}-1}f \biggl(\frac{ab}{ta+(1-t)b} \biggr)+t^{\frac {\alpha}{k}-1}f \biggl( \frac{ab}{(1-t)a+tb} \biggr)\leq t^{\frac{\alpha}{k}-1} \bigl[h(1-t)+h(t) \bigr] \bigl[f(a)+f(b) \bigr]. \end{aligned}$$

Integrating the above inequality with respect to *t* on $[0,1]$, we have

3.2 $$\begin{aligned} &{\biggl(\frac{ab}{b-a} \biggr)^{\frac{\alpha}{k}}k \Gamma_{k}(\alpha) \biggl\{ {}_{k}J_{\frac{1}{a^{-}}}^{\alpha}(f \circ g) \biggl(\frac{1}{b} \biggr) + {}_{k}J_{\frac{1}{b^{+}}}^{\alpha}(f \circ g) \biggl(\frac{1}{a} \biggr) \biggr\} } \\ &{\quad \leq \bigl[f(a)+f(b) \bigr] \int_{0}^{1}t^{\frac{\alpha}{k}-1} \bigl[h(1-t)+h(t) \bigr] \,\mathrm {d}{t}.} \end{aligned}$$

Summing inequalities (3.1) and (3.2) completes the proof. □

We now discuss some special cases of Theorem 3.1.

I. If $h(t)=t$ in Theorem 3.1, then we have the following new result.

### Corollary 3.2

*Let* $f:I\setminus\{0\}\rightarrow\mathbb{R}$ *be a harmonically convex function*, *where* $a,b\in I$ *with* $a< b$. *If* $f\in L[a,b]$, *then we have*

$$\begin{aligned} \frac{2k}{\alpha}f \biggl(\frac{2ab}{a+b} \biggr) \leq& \biggl(\frac{ab}{b-a} \biggr)^{\frac{\alpha}{k}}k\Gamma_{k}( \alpha) \biggl\{ {}_{k}J_{\frac{1}{a^{-}}}^{\alpha}(f\circ g) \biggl( \frac{1}{b} \biggr) + {}_{k}J_{\frac{1}{b^{+}}}^{\alpha}(f\circ g) \biggl(\frac{1}{a} \biggr) \biggr\} \\ \leq&\frac{k[f(a)+f(b)]}{\alpha}. \end{aligned}$$

II. If $h(t)=t^{s}$ in Theorem 3.1, then we have the following new result.

### Corollary 3.3

*Let* $f:I\setminus\{0\}\rightarrow\mathbb{R}$ *be a harmonically* *s*-*convex function*, *where* $a,b\in I$ *with* $a< b$. *If* $f\in L[a,b]$, *then we have*

$$\begin{aligned} \frac{2^{s}k}{\alpha}f \biggl(\frac{2ab}{a+b} \biggr) \leq& \biggl(\frac{ab}{b-a} \biggr)^{\frac{\alpha}{k}}k\Gamma_{k}( \alpha) \biggl\{ {}_{k}J_{\frac{1}{a^{-}}}^{\alpha}(f\circ g) \biggl( \frac{1}{b} \biggr) + {}_{k}J_{\frac{1}{b^{+}}}^{\alpha}(f\circ g) \biggl(\frac{1}{a} \biggr) \biggr\} \\ \leq& \bigl[f(a)+f(b) \bigr] \biggl(k B_{k} \bigl(\alpha,k(s+1) \bigr)- \frac{k}{\alpha+ks} \biggr). \end{aligned}$$

III. If $h(t)=t^{-s}$ in Theorem 3.1, then we have the following new result.

### Corollary 3.4

*Let* $f:I\setminus\{0\}\rightarrow\mathbb{R}$ *be a harmonically* *s*-*Godunova*-*Levin convex function*, *where* $a,b\in I$ *with* $a< b$. *If* $f\in L[a,b]$, *then*, *for* $\alpha>ks$, *we have*

$$\begin{aligned} \frac{k}{2^{s}\alpha}f \biggl(\frac{2ab}{a+b} \biggr) \leq& \biggl(\frac{ab}{b-a} \biggr)^{\frac{\alpha}{k}}k\Gamma_{k}( \alpha) \biggl\{ {}_{k}J_{\frac{1}{a^{-}}}^{\alpha}(f\circ g) \biggl( \frac{1}{b} \biggr) + {}_{k}J_{\frac{1}{b^{+}}}^{\alpha}(f\circ g) \biggl(\frac{1}{a} \biggr) \biggr\} \\ \leq& \bigl[f(a)+f(b) \bigr] \biggl(k B_{k} \bigl(\alpha,k(1-s) \bigr)- \frac{k}{\alpha-ks} \biggr). \end{aligned}$$

IV. If $h(t)=1$ in Theorem 3.1, then we have the following new result.

### Corollary 3.5

*Let* $f:I\setminus\{0\}\rightarrow\mathbb{R}$ *be a harmonic* *P*-*function*, *where* $a,b\in I$ *with* $a< b$. *If* $f\in L[a,b]$, *then we have*

$$\begin{aligned} \frac{k}{\alpha}f \biggl(\frac{2ab}{a+b} \biggr) \leq& \biggl(\frac{ab}{b-a} \biggr)^{\frac{\alpha}{k}}k\Gamma_{k}( \alpha) \biggl\{ {}_{k}J_{\frac{1}{a^{-}}}^{\alpha}(f\circ g) \biggl( \frac{1}{b} \biggr) + {}_{k}J_{\frac{1}{b^{+}}}^{\alpha}(f\circ g) \biggl(\frac{1}{a} \biggr) \biggr\} \\ \leq&\frac{2k[f(a)+f(b)]}{\alpha}. \end{aligned}$$

Now using the auxiliary results, we derive some trapezoidal and mid-point type inequalities.

### Theorem 3.6

*Assume that* $f:[0,1]\rightarrow\mathbb{R}$ *is a differentiable function such that* $\vert f^{\prime} \vert ^{q}$ *is a harmonic convex function on* $[0,1]$. *Then*

$$\begin{aligned} \bigl\vert T_{f}(a,b;\alpha,k;g) \bigr\vert \leq \frac{ab(b-a)}{2}\cdot I^{1-\frac{1}{q}}\cdot J^{\frac{1}{q}}, \end{aligned}$$

*where*

$$\begin{aligned} I= \int_{0}^{1}\frac{ \vert t^{\frac{\alpha}{k}}-(1-t)^{\frac{\alpha }{k}} \vert }{[ta+(1-t)b]^{2}}\,\mathrm {d}t = \frac{1}{b^{2}} \biggl(I_{1}-\frac{1}{2^{\alpha/k}}I_{2}+I_{3}-I_{4} \biggr), \end{aligned}$$

*with*

$$\begin{aligned} &{I_{1}=kF_{1,k} \biggl( k, -\alpha, 2k, 2k; \frac{1}{2k}, \frac{b-a}{2bk} \biggr) ;} \\ &{I_{2}=kB_{k}(\alpha+k,1)F_{1,k} \biggl( \alpha+k,0,2k,\alpha+k+1;0,\frac{b-a}{2bk} \biggr);} \\ &{I_{3}=kB_{k}(\alpha+k,1)F_{1,k} \biggl( \alpha+k,0,2k,\alpha+k+1;0,\frac{b-a}{bk} \biggr);} \\ &{I_{4}=kB_{k}(k,\alpha+k)F_{1,k} \biggl( k,0,2k, \alpha+2k; 0,\frac{b-a}{bk} \biggr),} \end{aligned}$$

*and*

$$\begin{aligned} J =& \int_{0}^{1}\frac{ \vert t^{\frac{\alpha}{k}}-(1-t)^{\frac{\alpha }{k}} \vert }{[ta+(1-t)b]^{2}} \biggl\vert f' \biggl(\frac{ab}{ta+(1-t)b} \biggr) \biggr\vert ^{q} \, \mathrm {d}t \\ \leq&\frac{1}{b^{2}} \biggl[ \bigl\vert f^{\prime}(a) \bigr\vert ^{q} \biggl(J_{1} -\frac{1}{2^{\alpha/k}}J_{2}+J_{5}-J_{7} \biggr) + \bigl\vert f^{\prime}(b) \bigr\vert ^{q} \biggl( \frac{1}{2}J_{3}-\frac{1}{2^{\alpha/k+1}}J_{4}+J_{6}-J_{8} \biggr) \biggr], \end{aligned}$$

*with*

$$\begin{aligned} &{J_{1}=kB_{k}(k,k)F_{1,k} \biggl( k,- \alpha-k,2k,2k; \frac{1}{2k},\frac{b-a}{2bk} \biggr);} \\ &{J_{2}=kB_{k}(\alpha+k,k)F_{1,k} \biggl(\alpha+ k,-k,2k,\alpha+2k; \frac{1}{2k},\frac{b-a}{2bk} \biggr);} \\ &{J_{3}=kB_{k}(2k,k)F_{1,k} \biggl( 2k,- \alpha,2k,3k; \frac{1}{2k},\frac{b-a}{2bk} \biggr);} \\ &{J_{4}=kB_{k}(\alpha+2k,k)F_{1,k} \biggl(\alpha+ 2k,0,2k,\alpha+3k;0,\frac{b-a}{2bk} \biggr);} \\ &{J_{5}=kB_{k}(\alpha+k,2k)F_{1,k} \biggl( \alpha+k,0,2k,\alpha+3k;0,\frac{b-a}{bk} \biggr);} \\ &{J_{6}=kB_{k}(\alpha+2k,k)F_{1,k} \biggl( \alpha+2k,0,2k,\alpha+3k;0,\frac{b-a}{bk} \biggr);} \\ &{J_{7}=kB_{k}(k,\alpha+2k)F_{1,k} \biggl( k,0,2k, \alpha+3k;0,\frac{b-a}{bk} \biggr);} \\ &{J_{8}=kB_{k}(2k,\alpha+k)F_{1,k} \biggl( 2k,0,2k,\alpha+3k;0,\frac{b-a}{bk} \biggr).} \end{aligned}$$

### Proof

From Lemma 2.1, using the property of modulus and the power-mean inequality, we have

$$\begin{aligned} &{\bigl\vert T_{f}(a,b;\alpha,k;g) \bigr\vert } \\ &{\quad= \biggl\vert \frac{ab(b-a)}{2} \int_{0}^{1}\frac{[t^{\frac{\alpha}{k}}-(1-t)^{\frac{\alpha }{k}}]}{[ta+(1-t)b]^{2}}f' \biggl(\frac{ab}{ta+(1-t)b} \biggr)\,\mathrm {d}t \biggr\vert } \\ &{\quad \leq\frac{ab(b-a)}{2} \int_{0}^{1}\frac{ \vert t^{\frac{\alpha}{k}}-(1-t)^{\frac{\alpha }{k}} \vert }{[ta+(1-t)b]^{2}} \biggl\vert f' \biggl(\frac{ab}{ta+(1-t)b} \biggr) \biggr\vert \,\mathrm {d}t} \\ &{\quad \leq\frac{ab(b-a)}{2}I^{1-\frac{1}{q}} J^{\frac{1}{q}},} \end{aligned}$$

where

3.3 $$\begin{aligned} &{I=\int_{0}^{1}\frac{ \vert t^{\frac{\alpha}{k}}-(1-t)^{\frac{\alpha }{k}} \vert }{[ta+(1-t)b]^{2}}\,\mathrm {d}t} \\ &{\quad =2 \int_{0}^{1/2}\frac{(1-t)^{\frac{\alpha}{k}}-t^{{\frac{\alpha}{k}}}}{ [ta+(1-t)b ] ^{2} }\,\mathrm {d}t+ \int_{0}^{1}\frac{t^{{\frac{\alpha}{k}}}-(1-t)^{\frac{\alpha}{k}}}{ [ta+(1-t)b ] ^{2} }\,\mathrm {d}t} \\ &{\quad =\frac{1}{b^{2}} \biggl[ I_{1}- \biggl( \frac{1}{2} \biggr) ^{\frac{\alpha}{k}}\cdot I_{2}+I_{3}-I_{4} \biggr],} \end{aligned}$$

with

$$\begin{aligned} &{I_{1}=\int_{0}^{1} \biggl(1-\frac{u}{2} \biggr) ^{\frac{\alpha}{k}} \biggl(1-\frac{b-a}{2b}u \biggr) ^{-2}\,\mathrm {d}u =kF_{1,k} \biggl( k, -\alpha, 2k, 2k; \frac{1}{2k}, \frac{b-a}{2bk} \biggr);} \\ &{I_{2}= \int_{0}^{1}u^{\frac{\alpha}{k}} \biggl(1- \frac{b-a}{2b}u \biggr) ^{-2}\,\mathrm {d}u} \\ &{\phantom{I_{2}} =kB_{k}(\alpha+k,1)F_{1,k} \biggl( \alpha+k,0,2k, \alpha+k+1;0,\frac{b-a}{2bk} \biggr);} \\ &{I_{3}= \int_{0}^{1}t^{\frac{\alpha}{k}} \biggl(1- \frac{b-a}{b}t \biggr) ^{-2}\,\mathrm {d}t} \\ &{\phantom{I_{3}=} =kB_{k}(\alpha+k,1)F_{1,k} \biggl( \alpha+k,0,2k, \alpha+k+1;0,\frac{b-a}{bk} \biggr);} \\ &{I_{4}= \int_{0}^{1}(1-t)^{\frac{\alpha}{k}} \biggl(1- \frac{b-a}{b}t \biggr) ^{-2}\,\mathrm {d}t =kB_{k}(k,\alpha+k)F_{1,k} \biggl( k,0,2k,\alpha+2k; 0, \frac{b-a}{bk} \biggr),} \end{aligned}$$

and using the harmonic convexity of $\vert f^{\prime} \vert ^{q}$, we have

3.4 $$\begin{aligned} J =& \int_{0}^{1}\frac{ \vert t^{\frac{\alpha}{k}}-(1-t)^{\frac{\alpha }{k}} \vert }{[ta+(1-t)b]^{2}} \biggl\vert f' \biggl(\frac{ab}{ta+(1-t)b} \biggr) \biggr\vert ^{q} \, \mathrm {d}t \\ \leq& \int_{0}^{1}\frac{ \vert t^{\frac{\alpha}{k}}-(1-t)^{\frac{\alpha }{k}} \vert }{[ta+(1-t)b]^{2}} \bigl[(1-t) \bigl\vert f^{\prime}(a) \bigr\vert ^{q}+t \bigl\vert f^{\prime}(b) \bigr\vert ^{q} \bigr] \,\mathrm {d}t \\ =&2 \int_{0}^{1/2}\frac{(1-t)^{\frac{\alpha}{k}}-t^{\frac{\alpha }{k}}}{[ta+(1-t)b]^{2}} \bigl[(1-t) \bigl\vert f^{\prime}(a) \bigr\vert ^{q}+t \bigl\vert f^{\prime}(b) \bigr\vert ^{q} \bigr] \,\mathrm {d}t \\ &{}+ \int_{0}^{1}\frac{t^{\frac{\alpha}{k}}-(1-t)^{\frac{\alpha }{k}}}{[ta+(1-t)b]^{2}} \bigl[(1-t) \bigl\vert f^{\prime}(a) \bigr\vert ^{q}+t \bigl\vert f^{\prime}(b) \bigr\vert ^{q} \bigr] \,\mathrm {d}t \\ =& \int_{0}^{1}\frac{ ( 1-\frac{u}{2} ) ^{\frac{\alpha}{k}}- (\frac{u}{2} ) ^{\frac{\alpha}{k}}}{[\frac{u}{2}a+ ( 1-\frac{u}{2} ) b]^{2}} \biggl[ \biggl( 1- \frac{u}{2} \biggr) \bigl\vert f^{\prime}(a) \bigr\vert ^{q}+\frac{u}{2} \bigl\vert f^{\prime}(b) \bigr\vert ^{q} \biggr] \,\mathrm {d}u \\ &{}+ \int_{0}^{1}\frac{t^{\frac{\alpha}{k}}-(1-t)^{\frac{\alpha }{k}}}{[ta+(1-t)b]^{2}} \bigl[(1-t) \bigl\vert f^{\prime}(a) \bigr\vert ^{q}+t \bigl\vert f^{\prime}(b) \bigr\vert ^{q} \bigr] \,\mathrm {d}t \\ =&\frac{1}{b^{2}} \biggl[ \bigl\vert f^{\prime}(a) \bigr\vert ^{q} \biggl(J_{1} -\frac{1}{2^{\alpha/k}}J_{2}+J_{5}-J_{7} \biggr) \\ &{}+ \bigl\vert f^{\prime}(b) \bigr\vert ^{q} \biggl( \frac{1}{2}J_{3}-\frac{1}{2^{\alpha/k+1}}J_{4}+J_{6}-J_{8} \biggr) \biggr], \end{aligned}$$

with

$$\begin{aligned} &{J_{1}= \int_{0}^{1} \biggl(1-\frac{1}{2}u \biggr) ^{\frac{\alpha}{k}+1} \biggl( 1-\frac{b-a}{2b}u \biggr) ^{-2}\,\mathrm {d}u =kB_{k}(k,k)F_{1,k} \biggl( k,-\alpha-k,2k,2k; \frac{1}{2k},\frac{b-a}{2bk} \biggr);} \\ &{J_{2}=\int_{0}^{1}u^{\frac{\alpha}{k}} \biggl(1- \frac{1}{2}u \biggr) \biggl( 1-\frac{b-a}{2b}u \biggr) ^{-2} \,\mathrm {d}u} \\ &{\phantom{J_{2}} =kB_{k}(\alpha+k,k)F_{1,k} \biggl(\alpha+ k,-k,2k, \alpha+2k; \frac{1}{2k},\frac{b-a}{2bk} \biggr);} \\ &{J_{3}= \int_{0}^{1}u \biggl(1-\frac{1}{2}u \biggr)^{\frac{\alpha}{k}} \biggl( 1-\frac{b-a}{2b}u \biggr) ^{-2} \,\mathrm {d}u =kB_{k}(2k,k)F_{1,k} \biggl( 2k,-\alpha,2k,3k; \frac{1}{2k},\frac{b-a}{2bk} \biggr);} \\ &{J_{4}= \int_{0}^{1}u^{\frac{\alpha}{k}+1} \biggl( 1- \frac{b-a}{2b}u \biggr) ^{-2}\,\mathrm {d}u =kB_{k}(\alpha+2k,k)F_{1,k} \biggl(\alpha+ 2k,0,2k, \alpha+3k;0,\frac{b-a}{2bk} \biggr);} \\ &{J_{5}= \int_{0}^{1}t^{\frac{\alpha}{k}} (1-t ) \biggl( 1- \frac{b-a}{b}t \biggr) ^{-2}\,\mathrm {d}t =kB_{k}(\alpha+k,2k)F_{1,k} \biggl( \alpha+k,0,2k, \alpha+3k;0,\frac{b-a}{bk} \biggr);} \\ &{J_{6}= \int_{0}^{1}t^{\frac{\alpha}{k}+1} \biggl( 1- \frac{b-a}{b}t \biggr) ^{-2}\,\mathrm {d}t =kB_{k}(\alpha+2k,k)F_{1,k} \biggl( \alpha+2k,0,2k, \alpha+3k;0,\frac{b-a}{bk} \biggr);} \\ &{J_{7}= \int_{0}^{1} (1-t )^{\frac{\alpha}{k}+1} \biggl( 1- \frac{b-a}{b}t \biggr) ^{-2}\,\mathrm {d}t =kB_{k}(k,\alpha+2k)F_{1,k} \biggl( k,0,2k,\alpha+3k;0, \frac{b-a}{bk} \biggr);} \\ &{J_{8}= \int_{0}^{1}t (1-t )^{\frac{\alpha}{k}} \biggl( 1- \frac{b-a}{b}t \biggr) ^{-2}\,\mathrm {d}t =kB_{k}(2k,\alpha+k)F_{1,k} \biggl( 2k,0,2k,\alpha+3k;0, \frac{b-a}{bk} \biggr),} \end{aligned}$$

and the proof is complete. □

### Theorem 3.7

*Assume that* $f:[0,1]\to\mathbb{R}$ *is a differentiable function such that* $\vert f^{\prime} \vert ^{q}$ *is a harmonic convex function on* [0,1]. *Then*

$$\begin{aligned} \bigl\vert M_{f}(a,b;\alpha,k;g) \bigr\vert \leq \frac{ab(b-a)}{2} \bigl(I^{1-1/q} J^{1/q}+K^{1-1/q}L^{1/q}+M^{1-1/q}N^{1/q} \bigr), \end{aligned}$$

*where* *I* *is given by* (3.3) , *J* *is given by* (3.4),

$$\begin{aligned} &{K=\frac{a}{b^{2}(a+b)},} \\ &{ L\leq\frac{ \vert f^{\prime}(a) \vert ^{q}}{2b^{2}}\cdot kB_{k}(k,k)F_{1,k} \biggl(k,-k,2k,2k;\frac{1}{2k},\frac{b-a}{2bk} \biggr)} \\ &{\phantom{L\leq} {} +\frac{ \vert f^{\prime}(b) \vert ^{q}}{4b^{2}}\cdot kB_{k}(2k,k)F_{1,k} \biggl(2k,0,2k,3k;0,\frac{b-a}{2bk} \biggr),} \\ &{M=\frac{1}{b(a+b)}} \end{aligned}$$

*and*

$$\begin{aligned} N \leq&\frac{ \vert f^{\prime}(a) \vert ^{q}}{2b^{2}} \biggl[F_{1,k} \biggl(k,0,2k,3k;0, \frac{b-a}{bk} \biggr)-F_{1,k} \biggl(k,-k,2k,2k;\frac{1}{2k}, \frac{b-a}{2bk} \biggr) \biggr] \\ &{} +\frac{ \vert f^{\prime}(b) \vert ^{q}}{2b^{2}} \biggl[F_{1,k} \biggl(2k,0,2k,3k;0, \frac{b-a}{bk} \biggr)-\frac{1}{4}F_{1,k} \biggl(2k,0,2k,3k;0, \frac{b-a}{bk} \biggr) \biggr]. \end{aligned}$$

### Proof

From Lemma 2.3, using the property of modulus and the power-mean inequality, we have

$$\begin{aligned} \bigl\vert M_{f}(a,b;\alpha,k;g) \bigr\vert \leq& \frac{ab(b-a)}{2} \biggl[ \int_{0}^{1}\frac{ \vert (1-t)^{\frac{\alpha}{k}}-t^{\frac{\alpha }{k}} \vert }{[ta+(1-t)b]^{2}} \biggl\vert f' \biggl(\frac{ab}{ta+(1-t)b} \biggr) \biggr\vert \,\mathrm {d}t \\ &{}+ \int_{0}^{\frac{1}{2}}\frac{1}{[ta+(1-t)b]^{2}} \biggl\vert f' \biggl(\frac{ab}{ta+(1-t)b} \biggr) \biggr\vert \,\mathrm {d}t \\ &{} + \int_{\frac{1}{2}}^{1}\frac{1}{[ta+(1-t)b]^{2}} \biggl\vert f' \biggl(\frac{ab}{ta+(1-t)b} \biggr) \biggr\vert \,\mathrm {d}t \biggr] \\ \leq&\frac{ab(b-a)}{2} \bigl(I^{1-1/q} J^{1/q}+K^{1-1/q}L^{1/q}+M^{1-1/q}N^{1/q} \bigr), \end{aligned}$$

where

$$\begin{aligned} K= \int_{0}^{\frac{1}{2}}\frac{1}{ [ta+(1-t)b ] ^{2} }\,\mathrm {d}t= \frac{a}{b^{2}(a+b)}, \end{aligned}$$

and

$$\begin{aligned} L&= \int_{0}^{\frac{1}{2}}\frac{1}{[ta+(1-t)b]^{2}} \biggl[ \biggl\vert f' \biggl(\frac{ab}{ta+(1-t)b} \biggr) \biggr\vert \biggr] ^{q} \,\mathrm {d}t \\ &\leq \int_{0}^{\frac{1}{2}}\frac{1}{[ta+(1-t)b]^{2}} \bigl[(1-t) \bigl\vert f^{\prime}(a) \bigr\vert ^{q}+t \bigl\vert f^{\prime}(b) \bigr\vert ^{q} \bigr]\,\mathrm {d}t, \end{aligned}$$

and using the change of variables, we have

$$\begin{aligned} &{L\leq \frac{1}{2b^{2}} \bigl\vert f^{\prime}(a) \bigr\vert ^{q} \int_{0}^{1} \biggl(1-\frac{1}{2}u \biggr) \biggl(1-u\cdot\frac{b-a}{2b} \biggr)^{-2}\,\mathrm {d}u} \\ &{\phantom{L\leq} {}+\frac{1}{4b^{2}} \bigl\vert f^{\prime}(b) \bigr\vert ^{q} \int_{0}^{1}u \biggl(1-u\cdot\frac{b-a}{2b} \biggr)^{-2}\,\mathrm {d}u} \\ &{\phantom{L} =\frac{ \vert f^{\prime}(a) \vert ^{q}}{2b^{2}}\cdot kB_{k}(k,k)F_{1,k} \biggl(k,-k,2k,2k;\frac{1}{2k},\frac{b-a}{2bk} \biggr)} \\ &{\phantom{L\leq} {} +\frac{ \vert f^{\prime}(b) \vert ^{q}}{4b^{2}}\cdot kB_{k}(2k,k)F_{1,k} \biggl(2k,0,2k,3k;0,\frac{b-a}{2bk} \biggr),} \\ &{ M= \int_{\frac{1}{2}}^{1}\frac{1}{[ta+(1-t)b]^{2}}\,\mathrm {d}t= \frac{1}{b(a+b)},} \end{aligned}$$

and

$$\begin{aligned} N =& \int_{\frac{1}{2}}^{1}\frac{1}{[ta+(1-t)b]^{2}} \biggl[ \biggl\vert f' \biggl(\frac{ab}{ta+(1-t)b} \biggr) \biggr\vert \biggr] ^{q} \,\mathrm {d}t \\ =& \int_{0}^{1}\frac{1}{[ta+(1-t)b]^{2}} \biggl[ \biggl\vert f' \biggl(\frac{ab}{ta+(1-t)b} \biggr) \biggr\vert \biggr] ^{q} \,\mathrm {d}t \\ &{}- \int_{0}^{\frac{1}{2}}\frac{1}{[ta+(1-t)b]^{2}} \biggl[ \biggl\vert f' \biggl(\frac{ab}{ta+(1-t)b} \biggr) \biggr\vert \biggr] ^{q} \,\mathrm {d}t \\ \leq&\frac{1}{b^{2}} \int_{0}^{1} \biggl( 1-t\cdot\frac{b-a}{b} \biggr) ^{-2} \bigl[(1-t) \bigl\vert f^{\prime}(a) \bigr\vert ^{q} +t \bigl\vert f^{\prime}(b) \bigr\vert ^{q} \bigr]\,\mathrm {d}t \\ &{}- \frac{1}{b^{2}} \int_{0}^{\frac{1}{2}} \biggl( 1-t\cdot\frac{b-a}{b} \biggr) ^{-2} \bigl[(1-t) \bigl\vert f^{\prime}(a) \bigr\vert ^{q} +t \bigl\vert f^{\prime}(b) \bigr\vert ^{q} \bigr]\,\mathrm {d}t \\ =&\frac{ \vert f^{\prime}(a) \vert ^{q}}{b^{2}} \biggl[ \int_{0}^{1}(1-t) \biggl( 1-t\cdot \frac{b-a}{b} \biggr) ^{-2}\,\mathrm {d}t- \int_{0}^{\frac{1}{2}}(1-t) \biggl( 1-t\cdot \frac{b-a}{b} \biggr) ^{-2}\,\mathrm {d}t \biggr] \\ &{}+\frac{ \vert f^{\prime}(b) \vert ^{q}}{b^{2}} \biggl[ \int_{0}^{1}t \biggl( 1-t\cdot\frac{b-a}{b} \biggr) ^{-2}\,\mathrm {d}t- \int_{0}^{\frac{1}{2}}t \biggl( 1-t\cdot\frac{b-a}{b} \biggr) ^{-2}\,\mathrm {d}t \biggr] \\ =&\frac{ \vert f^{\prime}(a) \vert ^{q}}{2b^{2}} \biggl[F_{1,k} \biggl(k,0,2k,3k;0, \frac{b-a}{bk} \biggr) -F_{1,k} \biggl(k,-k,2k,2k;\frac{1}{2k}, \frac{b-a}{bk} \biggr) \biggr] \\ &{}+\frac{ \vert f^{\prime}(b) \vert ^{q}}{2b^{2}} \biggl[F_{1,k} \biggl(2k,0,2k,3k;0, \frac{b-a}{bk} \biggr) -\frac{1}{4}F_{1,k} \biggl(2k,0,2k,3k;0,\frac{b-a}{2bk} \biggr) \biggr]. \end{aligned}$$

This completes the proof. □

## Conclusion

A new refinement of Hermite-Hadamard’s inequality via *k*-Riemann-Liouville fractional integrals is obtained. We have derived two new *k*-fractional integral identities. Utilizing these identities, we have derived some new *k*-fractional bounds which involve *k*-Appell’s hypergeometric functions via the functions which have the harmonic convexity property. It is expected that the ideas and techniques of this article may be useful for future research.
